# Serum Soluble Fms-Like Tyrosine Kinase 1 (sFlt-1) Predicts the Severity of Acute Pancreatitis

**DOI:** 10.3390/ijms17122038

**Published:** 2016-12-06

**Authors:** Paulina Dumnicka, Mateusz Sporek, Małgorzata Mazur-Laskowska, Piotr Ceranowicz, Marek Kuźniewski, Ryszard Drożdż, Tadeusz Ambroży, Rafał Olszanecki, Beata Kuśnierz-Cabala

**Affiliations:** 1 Department of Medical Diagnostics, Jagiellonian University Medical College, 30-688 Kraków, Poland; paulina.dumnicka@uj.edu.pl (P.D.); ryszard.drozdz@uj.edu.pl (R.D.); 2 Surgery Department, The District Hospital, 34-200 Sucha Beskidzka, Poland; msporek1983@gmail.com; 3 Department of Anatomy, Jagiellonian University Medical College, 31-034 Kraków, Poland; 4 Department of Diagnostics, University Hospital, 31-501 Kraków, Poland; mbmazur@cyf-kr.edu.pl; 5 Department of Physiology, Jagiellonian University Medical College, 31-531 Kraków, Poland; 6 Chair and Department of Nephrology, Jagiellonian University Medical College, 31-501 Kraków, Poland; marek.kuzniewski@uj.edu.pl; 7 Department of Theory of Sport and Kinesiology, Faculty of Physical Education and Sport, University of Physical Education, 31-571 Kraków, Poland; tadek@ambrozy.pl; 8 Department of Pharmacology, Jagiellonian University Medical College, 31-531 Kraków, Poland; mfolszan@cyf-kr.edu.pl; 9 Department of Diagnostics, Chair of Clinical Biochemistry, Jagiellonian University Medical College, 31-501 Kraków, Poland; mbkusnie@cyf-kr.edu.pl

**Keywords:** vascular endothelial growth factor receptor 1, acute pancreatitis, endothelial dysfunction, angiopoietin-2, diagnostic utility

## Abstract

Organ failure is the most important determinant of the severity of acute pancreatitis (AP). Soluble fms-like tyrosine kinase 1 (sFlt-1) is positively associated with organ failure in sepsis. Our aim was to evaluate the diagnostic utility of automated sFlt-1 measurements for early prediction of AP severity. Adult patients (66) with AP were recruited, including 46 with mild (MAP), 15 with moderately-severe (MSAP) and 5 with severe AP (SAP). Serum and urine samples were collected twice. Serum sFlt-1 was measured with automated electrochemiluminescence immunoassay. Serum concentrations of sFlt-1 were significantly higher in patients with MSAP and SAP as compared to MAP. SAP patients had the highest concentrations. At 24 and 48 h, sFlt-1 positively correlated with inflammatory markers (leukocyte count, C-reactive protein), kidney function (creatinine, urea, cystatin C, serum and urine neutrophil gelatinase-associated lipocalin, urine albumin/creatinine ratio), D-dimer and angiopoietin-2. sFlt-1 positively correlated with the bedside index of severity in AP (BISAP) score and the duration of hospital stay. Serum sFlt-1 above 139 pg/mL predicted more severe AP (MSAP + SAP). In the early phase of AP, sFlt-1 is positively associated with the severity of AP and predicts organ failure, in particular kidney failure. Serum sFlt-1 may be a practical way to improve early assessment of AP severity.

## 1. Introduction

Acute pancreatitis (AP) is the leading cause of hospital admissions due to gastrointestinal diseases in developed countries [1,2]. In most patients, the disease is mild; however, up to 20% develop the severe form, associated with persistent organ failure and high mortality [3]. According to current knowledge, as reflected by the Atlanta classification revised in 2012, the development of organ failure in the course of AP is the main factor determining the severity of the disease and related mortality [3]. Systemic inflammatory response syndrome (SIRS), diffuse endothelial activation and dysfunction and microcirculatory disorders are involved in the pathogenesis of organ failure in acute conditions, including AP [4,5,6].

Angiopoietin-2 (Ang-2), associated with endothelial dysfunction and vascular leakage in acute states [7], has been recently proposed as a marker of severity in AP [8,9,10]. Other markers of endothelial activation and dysfunction were shown to be increased in severe AP, including soluble E-selectin, tissue factor or von Willebrand factor and endothelial-specific microRNAs [5,11,12,13].

Fms-like tyrosine kinase-1 (Flt-1) is a membrane receptor binding vascular endothelial growth factor (VEGF)-A and -B, as well as placental growth factor (PlGF). It is also known as VEGF receptor-1 (VEGFR-1). Alternative splicing of Flt-1 precursor mRNA leads to the production of the soluble form of the receptor (sFlt-1) that acts as a decoy receptor to VEGF and PlGF [14]. VEGF is a potent stimulator of vascular permeability (for this reason, it was first named vascular permeability factor) [15]. Severe endothelial dysfunction observed in sepsis is associated with high concentrations of both VEGF and sFlt-1 in blood, and sFlt-1 is significantly correlated with the severity of organ dysfunction in sepsis patients [16]. VEGF has been implicated in the pathogenesis of experimental AP in rats [17,18,19], and high VEGF concentrations have been observed in plasma of patients with AP [20,21]; but very little is known about sFlt-1 concentrations in the course of AP.

We hypothesized that sFlt-1 may also be associated with AP severity. A practical advantage of this marker over the previously-mentioned Ang-2 or E-selectin is that it may be rapidly measured using routine automated analyzers. The automated assay to measure sFlt-1 has been developed and positively validated for use in the assessment of preeclampsia in pregnant women [22].

The aim of the study was to assess serum concentrations of sFlt-1 among patients with AP of various severity at the early phase of the disease (first 48 h from the onset of abdominal pain) and to evaluate the diagnostic utility of automated sFlt-1 measurements for the prediction of AP severity.

## 2. Results

Overall, 66 patients (34 men and 32 women) were included in the study. Among them, mild AP (MAP) was diagnosed in 46, moderately-severe AP (MSAP) in 15 and severe AP (SAP) in 5. Three deaths occurred in the studied group, all in the late phase of the disease (after 13–31 days of hospital stay). Because of the low number of patients with SAP, the data are reported together for MSAP and SAP patients (further referred to as MSAP + SAP). We have verified that the addition of SAP patients did not significantly change the MSAP group.

MAP patients did not differ significantly from those with more severe AP (MSAP + SAP) regarding age, sex, etiology, preexisting comorbid conditions and the duration of abdominal pain before admission (Table 1). As expected, all clinical characteristics related to the severity of the disease were significantly worse in MSAP + SAP group, resulting in more intensive treatment and a longer hospital stay (Table 1).

The MSAP + SAP group was characterized by higher concentrations of C-reactive protein (CRP), glucose, markers of renal function: creatinine, urea, cystatin C, urine albumin/creatinine ratio (uACR), serum and urine neutrophil gelatinase-associated lipocalin (NGAL), and D-dimer (Table 2). Furthermore, on the second day of AP (48 h from the onset of symptoms), leukocyte counts and amylase activity were higher in this group, while albumin and calcium concentrations were lower (Table 2). Serum concentrations of the endothelial markers, Ang-2 and sFlt-1 were higher in patients with MSAP and SAP as compared to MAP, both at 24 and 48 h from the onset of AP (Table 2). In particular, SAP patients had the highest sFlt-1:198 (183–213) pg/mL on the first day (*p* = 0.042 in comparison with the rest of the cohort). However, sFlt-1 significantly decreased after 48 h as compared to the first day of AP, both in patients with MAP (*p* = 0.003) and MSAP + SAP (*p* = 0.018). This was not observed in the case of Ang-2.

During the study, serum sFlt-1 positively correlated with inflammatory markers (leukocyte count, CRP), the markers of kidney function (serum creatinine, urea, cystatin C, serum and urine NGAL, uACR), as well as with the concentrations of D-dimer and Ang-2 (Table 3). Furthermore, sFlt-1 positively correlated with glucose on the first day of AP and negatively with albumin and calcium on the second day (Table 3).

Serum concentrations of sFlt-1 measured within the first 24 h from the onset of AP significantly predicted the severity of the disease, in particular the development of transient or persistent organ failure, both in simple analysis and after adjustment for age and the presence of comorbidities (Table 4; Appendix A). Although sFlt-1 was significantly positively correlated with serum creatinine and cystatin C, the association between sFlt-1 and more severe AP (MSAP + SAP) was independent of the markers of glomerular filtration (Table 5). CRP and sFlt-1 measured on the first day of AP were independent predictors of MSAP + SAP (Table 5).

**Figure 1:**
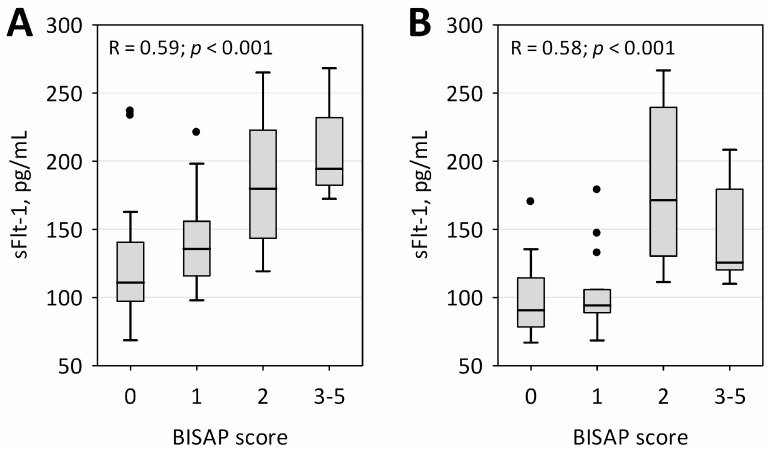
Correlation of Flt-1 serum concentrations with BISAP score during the first 24 (**A**) and at 48 h (**B**) from the onset of AP. Data are shown as the median, interquartile range (boxes), non-outlier range (whiskers) and outliers (points). Spearman correlation coefficients and p-values are shown on the graphs.

In both measurements, sFlt-1 concentrations were correlated with bedside index of severity in acute pancreatitis (BISAP) score; however, a more clear association of higher sFlt-1 with a higher BISAP score was observed within the first 24 h of AP (Figure 1). Furthermore, sFlt-1 on both days significantly positively correlated with the duration of hospital stay (R = 0.50; *p* < 0.001 on the first day and R = 0.45; *p* = 0.001 on the second day of AP).

**Figure 2:**
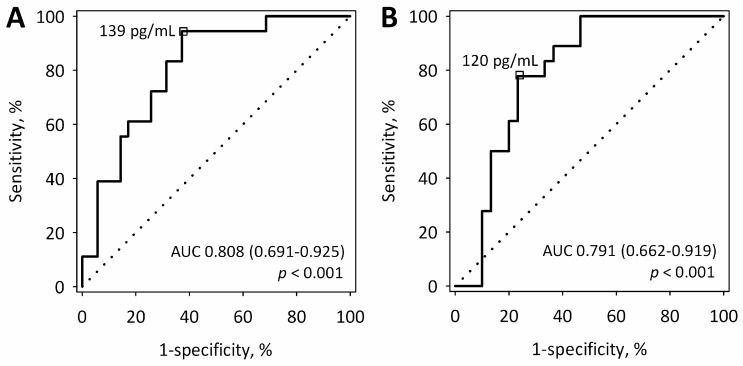
Receiver operating characteristic (ROC) curves for serum sFlt-1 measured within 24 (**A**) and at 48 h (**B**) from the onset of AP in the prediction of more severe acute pancreatitis (MSAP + SAP). The selected cut-off values are highlighted, and the values of area under the ROC curve (AUC) with 95% confidence intervals and *p*-values for the difference of AUC from AUC = 0.5 are shown on the graphs. The diagonal lines are the lines of no-discrimination.

On the first day of AP, serum sFlt-1 above 139 pg/mL predicted more severe AP (MSAP + SAP) with sensitivity of 94% and specificity of 63% (Figure 2A). On the second day, sFlt-1 above 120 pg/mL predicted MSAP + SAP with a sensitivity of 78% and a specificity of 77% (Figure 2B).

**Figure 3:**
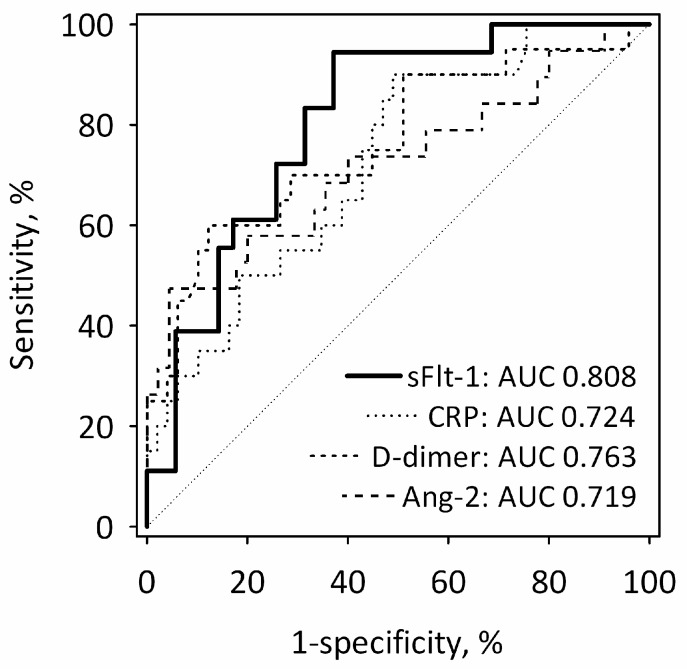
ROC curves for serum sFlt-1 measured within 24 h from the onset of AP in the prediction of more severe acute pancreatitis (MSAP + SAP) in comparison to other laboratory tests associated with AP severity. The values of AUC for each test are shown on the graph. The diagonal line is the line of no-discrimination.

On the first day of AP, the diagnostic utility of sFlt-1 for the prediction of MSAP + SAP was comparable with other single laboratory markers of AP severity, i.e., CRP, D-dimer and Ang-2 (Figure 3). The value of the area under the receiver operating characteristic (ROC) curve (AUC) was highest for sFlt-1, although it did not differ significantly from other markers’ AUCs. The combinations of single markers (sFlt-1 + CRP; sFlt-1 + D-dimer; sFlt-1 + Ang-2) did not predict MSAP + SAP significantly better than sFlt-1 alone.

**Figure 4:**
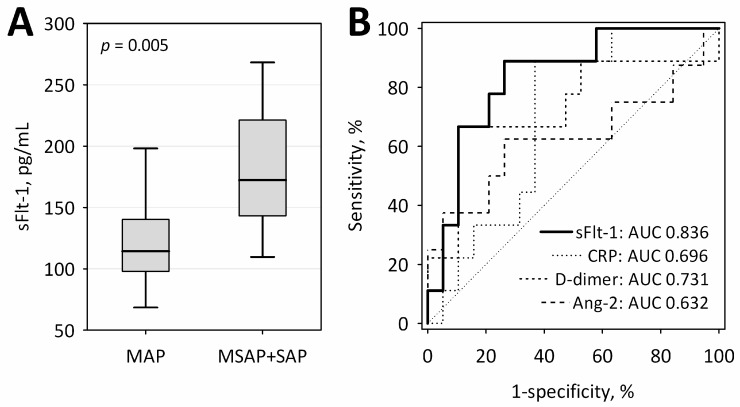
Serum concentrations of sFlt among 28 patients in whom blood samples were drawn at the earliest time point (18–21 h after the onset of AP symptoms), including 19 with MAP and nine who subsequently developed more severe AP (**A**); Data are shown as the median, interquartile range (boxes) and range (whiskers). The ROC curve for serum sFlt-1 measured at 18–21 h from the onset of AP in the prediction of more severe acute pancreatitis (MSAP + SAP) in comparison to other laboratory tests associated with AP severity (**B**). The values of AUC for each test are shown on the graph. The diagonal line is the line of no-discrimination.

Our intension was to collect the first blood sample within the first 24 h from the onset of pain due to AP at the time points close to the 24-h deadline. However, in 19 patients with MAP and nine patients who subsequently developed more severe AP, the first blood samples were collected between 18 and 21 h from the onset of AP symptoms (in the rest of the patients, samples were collected between 22 and 24 h) (Figure 4A). When we restricted the analysis to the 28 patients with samples drawn at the earliest time points, the estimate of AUC for sFlt-1 in the prediction of MSAP + SAP was even higher (AUC = 0.836; 95% confidence interval 0.680–0.992; *p* < 0.001 versus AUC = 0.5) (Figure 4B).

## 3. Discussion

In the report, we show the positive association between sFlt-1 measured in the sera of patients with AP on the first and second day of the disease evolution and the severity of the disease. Within the first 24 h of AP, the concentrations of sFlt-1 were highest in patients with SAP and enabled predicting a more severe course of the disease (MSAP + SAP, the development of organ failure or SIRS). Furthermore, sFlt-1 concentrations correlated with BISAP score, as well as CRP and D-dimer concentrations, recognized as predictors of severity and mortality in AP [23,24,25,26]. Moreover, sFlt-1 positively correlated with the length of hospital stay. On the first day of AP, sFlt-1 predicted more severe disease with high sensitivity and reasonable specificity. The diagnostic accuracy of sFlt-1 to predict more severe AP was comparable to other single markers of AP severity, including Ang-2. Recently, high diagnostic utility was reported for early Ang-2 measurements (AUCs of 0.940 and 0.851) in the prediction of SAP [8,9].

To our best knowledge, this is the first report where sFlt-1 was measured with an automated method in patients with AP. We could identify only one previous report on sFlt-1 serum concentrations among patients with AP. In a study of Espinosa et al. [27], including 25 patients with AP, serum sFlt-1 (or VEGFR-1) was measured with the enzyme-linked immunosorbent assay. The authors did not find higher concentrations of VEGFR-1 among seven patients with predicted severe AP, nor among seven patients with unfavorable clinical evolution of AP (defined as kidney, respiratory or cardiovascular failure, or local infectious or necrotic complications). However, they were able to find an association between Ang-2 serum concentrations and AP severity [27]. The discrepancy between our findings and those of Espinosa et al. may be a result of different methods of measurement or may probably be due to different ways of sample collection and handling (Espinosa et al. only say they used serum samples, with no further details). Moreover, Espinosa et al. collected blood samples at 12 h and five days after the admission of patients, and their patients were included up to 72 h from the onset of symptoms of AP; thus, the time points of blood collection were apparently different than in our study. In our study, the difference in sFlt-1 concentrations between patients with MAP and those with more severe AP was most significant at the earliest time points. Thus, it is possible that at later time points (at 72 h from the onset of AP and later), the association between sFlt-1 concentrations and AP severity becomes weaker. Our study was designed to assess sFlt-1 as an early marker of AP severity, as this is most relevant to clinical practice. Therefore, more studies are needed to explain the discrepancy between the results of Espinosa et al. [27] and ours.

Increased concentrations of sFlt-1 have been shown in sepsis and have been positively associated with more severe sepsis [16,28,29,30,31]. In 2010, Shapiro et al. [16] reported a strong association between sFlt-1 plasma concentrations and the severity of sepsis, as well as the development of organ dysfunction in sepsis. High concentrations of sFlt-1 were observed in patients with clinically-diagnosed sepsis and septic shock already at admission to the emergency department and were positively correlated with Acute Physiology and Chronic Health Evaluation II (APACHE II) and Sequential Organ Failure Assessment (SOFA) scores [30,31]. In the study of Skibstead et al. [30], sFlt-1 was the best predictor of organ dysfunction and mortality in sepsis among several markers of endothelial dysfunction. It is disputable whether endothelial dysfunction in patients with a severe course of AP may be comparable to the well-documented severe endothelial impairment observed in sepsis. There are only a few studies directly comparing such patients. In a small study of Hynninen et al. [32], nine patients with severe acute pancreatitis were compared with 11 patients with severe sepsis. In both groups, mortality was about 30%. Furthermore, in both groups, similar plasma concentrations of E-selectin were observed in serial measurements during the first three days following admission. At admission, E-selectin levels were significantly correlated with SOFA scores. E-selectin is expressed on activated endothelial cells; the soluble form is a result of the shedding of this membrane protein. Thus, Hynninen et al. [32] results suggest that similar activation of at least some signaling pathways of endothelial cells is associated with SAP and sepsis. On the other hand, sFlt-1 plasma concentrations were higher in patients with hypotension due to sepsis than in emergency department patients with non-sepsis hypotension of cardiac or hemorrhagic cause (median 227 versus 136 pg/mL); however, this study did not include patients with AP [33].

In the study of Shapiro et al. [16], the concentrations of sFlt-1 in patients with severe sepsis (median concentrations about 200 pg/mL) and septic shock (above 300 pg/mL) were higher than in our patients, including those with SAP (median concentrations 128 pg/mL in MAP, 161 pg/mL in MSAP and 198 pg/mL SAP on the first day). On the other hand, Skibstead et al. [30] reported median concentration of 168 pg/mL in patients with sepsis, comparable with our MSAP and SAP patients. However, we cannot directly compare the measured concentrations, as the measurements were done with different methods. Both Shapiro et al. [16] and Skibstead et al. [30] used a commercially available enzyme immunoassay and EDTA-plasma. The type of sample, as well as the administration of heparin as a part of the patients’ treatment have been shown to significantly affect the concentrations of sFlt-1 [34]. We have measured sFlt-1 concentrations in sera obtained from venous blood. Importantly, the assay we used is specifically dedicated to measure sFlt-1 in serum.

Except for sepsis, other acute conditions have also been associated with elevated levels of sFlt-1. Hochholzer et al. [35] measured sFlt-1 in sera of patients with suspected acute myocardial infarction and found increasing concentrations in those with unstable angina, non-ST-segment-elevation myocardial infarction and ST-elevation myocardial infarction. Notably, the study utilized the same method of measurements as ours. In patients without acute coronary syndrome, median sFlt-1 was about 70 pg/mL, while in those with ST-elevation myocardial infarction about 90 pg/mL [35]. In another study, higher sFlt-1 significantly predicted acute severe heart failure associated with myocardial infarction [34]. Furthermore, higher sFlt-1 was observed in patients who developed acute respiratory distress syndrome in the course of sepsis or trauma and following cardiac arrest [36].

In our study, sFlt-1 positively correlated with Ang-2 (although the correlation is of moderate strength), and as we have previously shown for Ang-2 [10], it was also significantly positively correlated with the markers of kidney function (including creatinine, urea, cystatin C, uACR, uNGAL and sNGAL) and predicted kidney failure. sFlt-1 has been shown to contribute in endothelial dysfunction to chronic kidney disease [37] and to correlate with mortality in patients on maintenance hemodialysis [38]. Furthermore, inhibition of VEGF signaling in renal glomeruli due to increased sFlt-1 has been implicated in the pathophysiology of kidney impairment and proteinuria observed in preeclampsia [39,40]. Interestingly, in our AP patients, sFlt-1 positively correlated with albuminuria (uACR). Kidney failure is among the most common organ complications of AP, observed in 16% of fatal cases [41]. We have previously observed that uNGAL concentrations predict the development of acute kidney injury in the course of AP [42]. Currently, there are possibilities to measure both uNGAL and sFlt-1 using routine automated laboratory methods. Simultaneous use of both markers may allow for early and reliable identification of patients at risk of acute kidney injury complicating AP. Importantly, although serum sFlt-1 concentrations were significantly correlated in our group with the markers of reduced glomerular filtration (serum creatinine and cystatin C), sFlt-1 predicted more severe AP (MSAP + SAP) independently of these markers. We may conclude that, although renal failure might have contributed to the increase in sFlt-1 observed in our patients, it definitely was not the single factor responsible for high sFlt-1 concentrations in more severe disease.

The design of our study does not allow drawing conclusions about the pathophysiological role of increased sFlt-1 in AP. Excerpt for endothelial cells, monocytes seem to be the important source of sFlt-1 in inflammatory conditions [43]. Nonetheless, in sepsis, sFlt-1 correlated significantly with recognized markers of endothelial dysfunction such as E-selectin or PAI-1 [30]. High sFlt-1 concentrations in sepsis and related conditions may reflect a protective response against increased VEGF, an endogenous compensatory anti-inflammatory mechanism [16,44]. In experimental sepsis in mice, endogenous sFlt-1 increased, whereas treatment with exogenous sFlt-1 attenuated the inflammatory response and endothelial dysfunction [44].

The limitation of our study is the low number of patients, especially those with SAP. For this reason, we were not able to reliably assess the diagnostic utility of sFlt-1 for the prediction of SAP. Nonetheless, we were able to show that sFlt-1 measured with the automated assay is positively associated with the severity of the disease and is an early predictor of organ failure, in particular kidney failure. We may hypothesize that the diagnostic utility of sFlt-1 might be better in patients’ groups including more SAP patients. If this is confirmed in further studies, serum sFlt-1 measured at admission may become a practical way to improve early assessment of AP severity, considering the availability of automated methods of sFlt-1 measurement. In this aspect, our results are promising, and we believe they ought to be validated in a larger cohort of AP patients, including more patients with SAP.

## 4. Methods

### 4.1. Patients and Study Protocol

We used frozen serum samples obtained in a prospective observational study that recruited consecutive patients diagnosed with AP, admitted and treated in the Surgery Department of the District Hospital in Sucha Beskidzka, Poland. AP was diagnosed according to the 2012 revision of the Atlanta Classification, i.e., when at least two of the following features were present: abdominal pain consistent with AP, serum amylase activity above three-times greater than the upper reference limit and characteristic findings of AP on abdominal imaging (contrast-enhanced computer tomography, magnetic resonance imaging or transabdominal ultrasonography) [3]. Only adult patients who gave written informed consent for the study were included. Patients who were admitted later that 24 h from the onset of pain due to AP were excluded. Furthermore, patients with chronic pancreatitis, chronic liver diseases (cirrhosis or viral hepatitis), diagnosed neoplasms of any origin or those treated with anticoagulants (including heparin in any form) were excluded. During the first 48 h from the onset of pain due to AP (i.e., when the blood samples were collected for the study), none of the patients received heparin or were dialyzed.

Demographic and clinical data were collected from patients at admission (age, sex, history of comorbidities, history of alcohol consumption, duration of pain until admission) and during the hospital stay (data regarding the course of AP, including development and duration of organ failure, development of local or systemic complications, treatment used, duration of hospital stay and outcome). The BISAP score was calculated using data collected during the first 24 h of AP [23]. Organ failure, including kidney failure, was diagnosed according to a modified Marshall scoring system, as cited in the revised Atlanta Classification [3].

Based on clinical evolution of AP, MAP, MSAP or SAP was diagnosed, in concordance with the revised Atlanta Classification [3]. MAP was defined as no organ failure, local or systemic complications during the hospital stay. MSAP was diagnosed when a patient presented transient organ failure (lasting less than 48 h), local (necrosis, acute necrotic collection, walled-off pancreatic necrosis) or systemic complications (exacerbation of preexisting conditions). SAP was diagnosed in patients with persistent organ failure (lasting more than 48 h).

Venous blood and urine samples for laboratory tests were collected from the patients twice, within the first 24 h (first day) and about 48 h (second day) from the onset of pain due to AP.

The study was conducted in accordance with the Declaration of Helsinki. The study protocol was approved by the Bioethics Committee of the Jagiellonian University (Approval No. KBET/247/B/2013, permission date 28th November 2013 and 122.6120.242.2015, permission date 22nd November 2015).

### 4.2. Laboratory Tests

Routine laboratory tests included complete blood counts performed in EDTA-anticoagulated whole blood, as well as the measurements of albumin, calcium, glucose, creatinine, urea and CRP concentrations in serum, amylase activity in serum and D-dimer concentrations in citrated plasma. These tests were done on the day of blood collection, with the use of automated analyzers, in the Department of Laboratory Diagnostics, District Hospital in Sucha Beskidzka, Poland.

Urinary concentrations of NGAL were measured on the day of urine collection, using chemiluminescent microparticle immunoassay and Architect analyzer (Abbott Diagnostics, Lake Forest, IL, USA), in the Department of Laboratory Diagnostics, District Hospital in Sucha Beskidzka, Poland. Aliquots of urine were frozen in −70 °C and further used to measure urinary albumin and creatinine. Urinary albumin was measured by immunonephelometry and urinary creatinine by the Jaffe method on automated analyzers in the Diagnostic Department, University Hospital, Kraków, Poland. The results of these measurements were expressed as the urine albumin to creatinine ratio (uACR).

Serum samples for measurements of serum NGAL, cystatin C, Ang-2 and sFlt-1 were processed according to standard procedure, i.e., blood was collected from antecubital vein into standard serum tubes, allowed to fully clot for 30 min and centrifuged (10 min, 2000× *g*); serum was aliquoted and frozen in −70 °C (the whole procedure was completed within 1 hour from blood collection). The procedure was consistent with the instructions of the manufacturers of the laboratory assays used, including the sFlt-1 assay. Cystatin C was measured by immunonephelometry using the Nephelometer BN II analyzer (Siemens Healthcare, Erlangen, Germany), and sFlt-1 was measured by electrochemiluminescence immunoassay using the Cobas 8000 analyzer (Roche Diagnostics, Mannheim, Germany) in the Diagnostic Department, University Hospital, Kraków, Poland. The enzyme immunoassays were used to measure sNGAL and Ang-2, i.e., Human Lipocalin-2/NGAL ELISA (BioVendor, Brno, Czech Republic) and Quantikine ELISA Human Angiopoietin-2 (R&D Systems, Minneapolis, MN, USA), respectively. Enzyme immunoassays were performed in the Department of Diagnostics, Chair of Clinical Biochemistry, Jagiellonian University Medical College, Kraków, Poland.

### 4.3. Statistical Analysis

Data were shown as the number (percentage) for categories, the median (lower-upper quartile) for non-normally distributed quantitative variables and the mean ± standard deviation for normally-distributed quantitative variables. Distributions were tested for normality with the Shapiro–Wilk test. The chi-squared test, Mann–Whitney test and unpaired t-test were used to study the differences between groups, respectively. The Wilcoxon signed rank test was used to analyze differences in repeated measurements. The correlations of sFlt-1 were assessed using the Spearman rank correlation coefficient, as the distribution of sFlt-1 differed significantly from normal. Simple and multiple logistic regression adjusted for age and the presence of comorbidities (i.e., the variables recognized as important predictors of AP severity [45]) were calculated to evaluate sFlt-1 as a predictor of severity of AP. Furthermore, separate multiple logistic regression models were calculated in order to check whether sFlt-1 predicts AP severity independently of renal function (serum creatinine and cystatin C concentrations) and inflammatory marker (CRP). Receiver operating characteristic (ROC) curves were used to assess the diagnostic accuracy of sFlt-1. The tests were two-tailed, and the results were considered significant at *p* ≤ 0.05. The Statistica 12 software package (StatSoft, Tulsa, OK, USA) was used for computations.

## Figures and Tables

**Table 1:** Clinical characteristics of patients.

Characteristic	MAP (*n* = 46)	MSAP + SAP (*n* = 20)	*p*
Age, years	58 ± 19	66 ± 16	NS
Male sex, N (%)	25 (54)	9 (45)	NS
Etiology	–	–	–
Gallstone, N (%)	27 (59)	8 (40)	NS
Alcohol, N (%)	11 (24)	7 (35)	–
Hypertriglyceridemia, N (%)	3 (7)	2 (10)	–
Other, N (%)	5 (11)	3 (15)	–
Preexisting comorbidities, N (%)	33 (72)	18 (90)	NS
Hypertension, N (%)	14 (30)	9 (45)	–
Ischemic heart disease, N (%)	11 (24)	7 (35)	–
Diabetes, N (%)	5 (11)	5 (25)	–
Lung diseases, N (%)	4 (9)	3 (16)	–
Duration of pain until admission, hours	12 (6–24)	12 (12–24)	NS
Organ failure: transient/persistent, N (%)	0/0	7 (35)/5 (25)	<0.001
BISAP score during first 24 h	0 (0–1)	2 (1–3)	<0.001
≥3 points, N (%)	0	7 (35)	
SIRS, N (%)	0	9 (45)	<0.001
Pancreatic or peripancreatic necrosis, N (%)	0	3 (15)	0.025
Peripancreatic fluid collections, N (%)	0	5 (25)	0.002
Pleural effusion, N (%)	0	14 (70)	<0.001
Antibiotic prophylaxis or treatment, N (%)	15 (33)	17 (85)	<0.001
Parenteral nutrition, N (%)	0	3 (15)	0.025
Surgery, N (%)	0	3 (15)	0.025
Duration of hospital stay, days	6 (5–7)	12 (10–21)	<0.001
Early/late mortality, N (%)	0/0	0/3 (15)	0.025

Abbreviations: MAP, mild acute pancreatitis; MSAP, moderately-severe acute pancreatitis; SAP, severe acute pancreatitis; N, number of patients; BISAP, bedside index of severity in acute pancreatitis; SIRS, systemic inflammatory response syndrome; NS, non-significant.

**Table 2:** The results of selected laboratory tests within the first 24 and at 48 h from the onset of AP.

Variable	Time Point	MAP (*n* = 46)	MSAP + SAP (*n* = 20)	*p*
Hematocrit, %	24 h	42.5 ± 4.08	42.1 ± 6.91	NS
	48 h	39.6 ± 4.50	37.2 ±6.18	NS
Leukocyte count, ×10^3^/µL	24 h	11.2 (9.3–14.6)	11.2 (10.3–16.0)	NS
	48 h	8.6 (6.3–11.2)	14.6 (9.7–18.6)	0.001
Platelet count, ×10^3^/µL	24 h	235 ± 58	212 ± 75	NS
	48 h	209 ± 65	183 ± 80	NS
C-reactive protein, mg/L	24 h	6.5 (2.5–47.9)	74.6 (13.7–133.2)	0.003
	48 h	73.9 (32.1–142.8)	237.5 (161.8–299.0)	<0.001
Albumin, g/L	24 h	40.6 ± 4.23	37.1 ± 6.8	NS
	48 h	38.1 ± 3.23	31.4 ± 7.9	<0.001
Amylase, U/L	24 h	1076 (570–1648)	1031 (733–1917)	NS
	48 h	149 (105–251)	286 (142–478)	0.019
Calcium, mmol/L	24 h	2.33 ± 0.17	2.24 ± 0.29	NS
	48 h	2.28 ± 0.10	1.98 ± 0.26	<0.001
Glucose, mmol/L	24 h	7.70 (6.40–9.79)	9.18 (7.07–12.56)	0.038
	48 h	4.92 (4.53–5.73)	6.20 (5.52–8.07)	0.005
Creatinine, µmol/L	24 h	72.5 (63.1–94.8)	93.4 (72.6–165.3)	0.016
	48 h	69.2 (60.6–84.8)	85.8 (68.0–191.6)	0.020
Urea, mmol/L	24 h	5.37 (4.14–6.70)	7.24 (5.94–13.45)	0.003
	48 h	4.14 (3.33–5.04)	8.80 (3.49–15.78)	0.004
Cystatin C, mg/L	24 h	0.87 (0.65–1.07)	1.37 (0.79–1.78)	0.036
	48 h	0.82 (0.73–1.26)	1.60 (0.81–2.19)	0.008
uNGAL, µg/L	24 h	24.3 (14.6–37.3)	145 (72–670)	<0.001
	48 h	25.6 (15.0–46.0)	118 (71–293)	<0.001
sNGAL, µg/L	24 h	104 (64–139)	199 (116–276)	0.003
	48 h	137 (77–196)	250 (194–416)	<0.001
uACR, mg/g	24 h	28.8 (20.7–67.4)	87.2 (50.1–917.8)	0.011
	48 h	34.0 (19.8–84.6)	68.5 (29.7–94.5)	NS
D-dimer, µg/mL	24 h	1.54 (0.93–2.30)	3.70 (1.47–13.57)	0.001
	48 h	1.70 (1.06–2.17)	5.63 (3.24–11.37)	<0.001
Angiopoietin-2, ng/mL	24 h	2.89 (2.05–4.01)	4.29 (2.40–20.37)	0.006
	48 h	2.78 (1.91–4.24)	7.23 (3.69–15.18)	<0.001
sFlt-1, pg/mL	24 h	128 (104–163)	184 (143–223)	<0.001
	48 h	94 (85–119)	140 (120–179)	0.001

Abbreviations: see Table 1; uNGAL, urine neutrophil gelatinase-associated lipocalin; sNGAL, serum neutrophil gelatinase-associated lipocalin; uACR, urine albumin/creatinine ratio; sFlt-1, soluble fms-like tyrosine kinase 1; NS, non-significant.

**Table 3:** Correlations between sFlt-1 and selected laboratory results within the first 24 and at 48 h from the onset of AP.

Variable	24 h		48 h	
	R	*p*	R	*p*
Leukocyte count	0.49	<0.001	0.41	0.003
C-reactive protein	0.32	0.021	0.43	0.002
Albumin	−0.16	NS	−0.43	0.002
Glucose	0.34	0.011	0.12	NS
Calcium	−0.01	NS	−0.32	0.021
Creatinine	0.61	<0.001	0.42	0.002
Urea	0.54	<0.001	0.33	0.020
Cystatin C	0.67	<0.001	0.41	0.005
uNGAL	0.41	0.005	0.47	0.001
sNGAL	0.50	<0.001	0.65	<0.001
uACR	0.56	<0.001	0.32	0.022
D-dimer	0.36	0.008	0.36	0.008
Angiopoietin-2	0.38	0.006	0.37	0.008

Abbreviations: see Table 1; uNGAL, urine neutrophil gelatinase-associated lipocalin; sNGAL, serum neutrophil gelatinase-associated lipocalin; uACR, urine albumin/creatinine ratio; sFlt-1, soluble fms-like tyrosine kinase 1; NS, non-significant.

**Table 4:** The results of simple and multiple logistic regression to predict the severity of AP. Multiple models were adjusted for age and the presence of comorbidities.

Dependent Variable	Odds Ratio (95% Confidence Interval) per 10 pg/mL Increase in sFlt-1 Measured within 24 h from the Onset of AP; *p*-Value	
	Simple Analysis	Multiple Analysis ^1^
MSAP + SAP	1.28 (1.10–1.50); *p* = 0.001	1.30 (1.09–1.55); *p* = 0.003
BISAP ≥3 in the first 24 h	1.30 (1.07–1.59); *p* = 0.007	1.28 (1.04–1.59); *p* = 0.019
SIRS	1.27 (1.07–1.52); *p* = 0.006	1.30 (1.08–1.57); *p* = 0.007
Transient or persistent organ failure	1.44 (1.16–1.79); *p* < 0.001	1.41 (1.12–1.77); *p* = 0.003
Renal failure	1.31 (1.06–1.63); *p* = 0.010	1.31 (1.03–1.65); *p* = 0.022

^1^ The estimated odds ratios and *p*-values for the covariates in multiple models are presented in Appendix A (Table A1, Table A2, Table A3, Table A4 and Table A5). Abbreviations: see Table 1; sFlt-1, soluble fms-like tyrosine kinase 1.

**Table 5:** Multiple logistic regression to predict MSAP + SAP. The results of laboratory tests within the first 24 h of AP were used as predictor variables.

Independent Variables	Odds Ratio (95% Confidence Interval); *p*-Value		
	Model 1	Model 2	Model 3
sFlt-1, per 10 pg/mL	1.21 (1.02–1.42); *p* = 0.023	1.20 (1.01–1.44); *p* = 0.032	1.27 (1.08–1.50); *p* = 0.004
Serum creatinine, per 1 µmol/L	1.02 (1.00–1.04); *p* = 0.1	Not included	Not included
Serum cystatin C, per 1 mg/L	Not included	2.49 (0.56–11.01); *p* = 0.2	Not included
CRP, per 10 mg/L	Not included	Not included	1.12 (1.00–1.24); *p* = 0.041

Abbreviations: see Table 1; sFlt-1, soluble fms-like tyrosine kinase 1; CRP, C-reactive protein.

## Abbreviations

AP	acute pancreatitis
SIRS	systemic inflammatory response syndrome
Ang-2	angiopoietin-2
sFlt-1	soluble fms-like tyrosine kinase-1
VEGF	vascular endothelial growth factor
PlGF	placental growth factor
VEGFR-1	vascular endothelial growth factor receptor-1
BISAP	bedside index of severity in acute pancreatitis
MAP	mild acute pancreatitis
MSAP	moderately severe acute pancreatitis
SAP	severe acute pancreatitis
CRP	C-reactive protein
uNGAL	urinary neutrophil gelatinase-associated lipocalin
uACR	urine albumin to creatinine ratio
sNGAL	serum neutrophil gelatinase-associated lipocalin
ROC	receiver operating characteristic
AUC	area under receiver operating characteristic curve

## Appendix A

Multiple logistic regression models to predict the severity of acute pancreatitis are presented in Table A1, Table A2, Table A3, Table A4 and Table A5. Serum concentrations of sFlt-1 on Day 1 (within the first 24 h from the onset of pain), age and comorbidities were assessed as independent variables to predict the development of MSAP or SAP (Table A1), BISAP ≥ 3 (Table A2), SIRS (Table A3), transient or persistent organ failure (Table A4) and renal failure (Table A5).

**Table A1:** Multiple logistic regression model to predict MSAP + SAP.

Independent Variable	Odds Ratio (95% Confidence Interval)	*p*
sFlt-1 on Day 1, per 10 pg/mL	1.30 (1.09–1.55)	0.003
Age, per year	0.99 (0.95–1.03)	0.6
Presence of comorbidities	7.99 (0.69–92.80)	0.09
Whole model	not applicable	<0.001

**Table A2:** Multiple logistic regression model to predict BISAP ≥ 3 in the first 24 h.

Independent Variable	Odds Ratio (95% Confidence Interval)	*p*
sFlt-1 on Day 1, per 10 pg/mL	1.28 (1.04–1.59)	0.019
Age, per year	1.01 (0.94–1.08)	0.8
Presence of comorbidities	2.47 (0.30–20.70)	0.4
Whole model	not applicable	0.014

**Table A3:** Multiple logistic regression model to predict SIRS.

Independent Variable	Odds ratio (95% Confidence Interval)	*p*
sFlt-1 on Day 1, per 10 pg/mL	1.30 (1.08–1.57)	0.007
Age, per year	0.98 (0.93–1.03)	0.5
Presence of comorbidities	2.06 (0.17–24.71)	0.6
Whole model	not applicable	0.016

**Table A4:** Multiple logistic regression model to predict transient or persistent organ failure.

Independent Variable	Odds Ratio (95% Confidence Interval)	*p*
sFlt-1 on Day 1, per 10 pg/mL	1.41 (1.12–1.77)	0.003
Age, per year	1.04 (0.97–1.12)	0.2
Presence of comorbidities	1.04 (0.14–7.81)	0.9
Whole model	not applicable	<0.001

**Table A5:** Multiple logistic regression model to predict renal failure.

Independent Variable	Odds Ratio (95% Confidence Interval)	*p*
sFlt-1 on Day 1, per 10 pg/mL	1.31 (1.03–1.65)	0.022
Age, per year	1.03 (0.96–1.11)	0.4
Presence of comorbidities	0.39 (0.04–4.19)	0.4
Whole model	not applicable	0.018
